# Book Reviews

**Published:** 1916-01

**Authors:** 


### BOOK REVIEWS

PRACTICAL ORAL HYGIENE PROPHYLAXIS AND PYORRHEA ALVEOLARIS. By Robin Adair, B. S., M. D., D. D. S. Second Edition. Enlarged and revised. 462 pages and 124 illustrations. Oral Hygiene Publishnig Co., Atlanta, Ga. 1915. $5.00.

This work has three divisions. The first dealing with Practical Oral Hygiene, the second with Practical Oral Prophylaxis and the third with a discussion of Pyorrhea Alveolaris and the different methods of treating this disease. To the dentist, to the teacher and to the physician, the first part of this work should prove of great value since in it the author discusses the fundamental principles of Oral Hygiene from infancy to maturity. Lectures on Oral Hygiene are given in detail. They include contributions from such well-known dentists as Drs. Stevenson,

Albray, Zarbaugh, Corley, Hyatt, Hunt, and others. A lecture which should be especially commended is that issued by the Michigan Dental Society on the "Teeth and Their Care." This single lecture would repay one for the time spent in reading the book, especially one interested in the conservation of the health of school children. Think of it—only four children out of each hundred are not suffering from diseased teeth! In the latter part of this section are given numerous forms and outlines used by Boards of Health and School Clinics for the dental inspection of school children. There are valuable suggestions for the conduction of such clinics and many outlines which will prove of value in establishing school and industrial dental dispensaries.

The second part dealing with Oral Prophylaxis includes not only the views of the author, but also those of many of the most prominent and well-known dentists in America. As the great advances in medicine are being made in the prevention rather than the cure if disease, so in dentistry the modern tendency is toward prophylaxis. It is shown by the author that to carry out prophylaxis properly we should begin with the child and continue this all during life. In addition to showing the advantages of prophylaxis, the author gives various methods of carrying out this treatment. These include views of such dentists as Kelly, Goble, Johnson and others. The training and teaching of the dental nurse occupies a prominent place in this connection. Massachusetts already has a law regulating the practice of oral prophylaxis by a registered dental assistant. The teaching of oral prophylaxis and the practical methods employed by the author are discussed in detail.

The third section of the work is devoted to that most important of all mouth infections—Pyorrhea Alveolaris. The author discusses in detail the etiology, pathology, symptomatology and diagnosis of this disease. After this, the most approved methods of treating it are given. The views of Drs. Hartzell, Patterson, Sarrizan, Leroy, Fletcher, Meiseberger, Head, Merritt, Dunlop, Lundy, West and Reid are given as described by them especially for this work. Following these the author gives his own original method and system of treating Pyorrhea. The various complications and the methods of handling surgical procedures necessary and the results obtained from them are carefully described and discussed. A separate chapter is devoted to the use of autogenous vaccines and emetine hydrochloride in the treatment of the various pyorrheal conditions.

Every dentist, physician, teacher and layman who is interested in this field of work will find much of practical value in the text. For the teacher and physician the two first divisions of the work are most important, especially since Dr. Adair has recognized the important part which mouth infections play in their effect on the general health. He shows that both the medical and dental professions have an opportunity before them to assist in the preservation of the health of the community by realizing the importance of mouth hygiene. To accomplish the best results mouth hygiene should begin with the child. The dentists will find much of value in the detailed technical descriptions of methods of treatment and surgical operations in the various mouth infections.

The numerous illustrations, the excellent arrangement of subjects, the clear and simple style and the general make-up of the book make of it a most interesting and valuable work. A. H. B.

---

POST-MORTEM EXAMINATIONS. By William S. Wadsworth, M. D., Coroner's Physician of Philadelphia. Octavo volume of 598 pages with 304 original illustrations. Philadelphia and London: W. B. Saunders Company, 1915. Cloth, $6.00 net: Half-Morocco, $7.50 net.

The value of this work from a practical standpoint can be very easily recognized by those who have faced the problem of interpreting the findings correctly of postmortems. The large experience of Dr. Wadsworth in over four thousand examinations in which he has studied and systematized his findings renders his work an authority on the subject. As stated by him it is to follow the tendency to return to the study of the human body as a harmonized whole rather than a study of isolated parts that he has brought together the material in this work.

His manner of presentation is entirely new as he treats the subject from a practical rather than from a theoretical standpoint. The material from which Dr. Wadsworth draws his conclusion is what he has investigated and studied himself and many of his conclusions are entirely new. He has presented much matter that is not adequately discussed in other texts. Pathology is given a place of minor importance. The section devoted to medicolegal postmortems is of special value to all those interested in this phrase of work.

The illustrations, all of which are original, are most excellent and add greatly to the value and usefulness of the text. Taken as a whole this is undoubtedly the best and most practical work we have on this subject. A. H. B.

---

DIFFERENTIAL DIAGNOSIS—Volume II. Presented through an Analysis of 317 years By Richard C. Cabot, M. D., Assistant Professor of Clinical Medicine, Harvard Medical School. Octavo of 709 pages, 254 illustrations. Philadelphia and London: W. B. Saunders Company, 1914. Cloth $5.50; Half Morocco $7.00.

The second valume of Dr. Cabot's epoch-making work in Diagnosis is presented to the public and most heartily welcomed by everyone at all familiar with the first volume.

It follows the same plan of announcement of conditions and exhibition of cases illustrating the various phases of the general symptoms. It tells one what the things the patient complains of mean and shows the student and the worker how to begin to analyze and then to get hold of what is brought to him for treatment.

We used to be taught in college that a disease was a certain thing and had symptoms that showed its presence. Then the symptoms were given and they might mean almost anything unless the student were specially coached concerning them. Headache, Dyspepsia, Dizziness were common things to the student, they looked all alike as from time to time, he heard of them in connection with some disease the writer was trying his best to cover over with a lot of fine words.

Cabot takes the things the patient notices and holds them up before the student in every possible light so that he can see what they mean in their shadings and relations and thus find the way to diagnosis. Case after case has a series of symptoms outlined and correlated. Each one is "discussed" briefly and pointedly and tests indicated to determine the value of each complaint. Then when all has been finished, the "outcome" is told and if there has been error, it is shown just as if there has been success, it is indicated.

Take a few of the symptoms studied such as Abdominal and Other Tumors, Diarrhea, Dyspepsia, Hematemesis, Edema of the Legs, Hoarseness, Pallor, Palpitation and Arhythmia, Ascites and Abdominal Enlargement and the like. Take stock of what you know of these and what they might suggest to you when the patient comes telling of them. Unless you have read Cabot or grouped things in some such way as he does, you have been limited in all your work. He puts it all where you can get it and use it and feel sure that up to date there is nothing more to be looked for.

The book is authoritative and of the utmost value to every practitioner.

---

BONE-GRAFT SURGERY. By Fred H. Albee, M. D., F. A. C. S., Professor of Orthopedic Surgery at the New York Post-Graduate Medical School and the University of Vermont. Octavo volume of 417 pages with 332 illustrations, 3 of them in colors. Philadelphia and London: W. B. Saunders Company, 1915. Cloth $6.00 net; Half Morocco $7.50 net.

It is useless for one to expect to become efficient in any line of work if the fundamental principles of the work undertaken are not well understood. We are glad to see that the author devotes so much space to this consideration. The newer instruments and apparatus are given the consideration they deserve; for without the proper operating outfit, bone surgery cannot be properly done. Make-shifts are easier in almost any other branch of surgery.

Every department of bone surgery in which a graft can be used is well covered in this book. The illustrations are ample and instructive. The author has shown that there is scarcely a fracture where an inlay graft or a bone peg cannot be used, thus doing away with foreign material which has so often required removal. Kangaroo tendon is advocated instead of silver wire, so that there will be nothing in contact with the bone that cannot be utilized. We are glad to recommend Dr. Albee's book to any one undertaking bone-graft surgery. J. L. C.

---

BANDAGING. By A. D. Whiting, M. D., Instructor in Surgery at the University of Pennsylvania. 12mo of 151 pages, with 117 original illustrations. Philadelphia and London: W. B. Saunders Company, 1915. Cloth, $1.25 net.

Not so many years ago, the surgeon took a deal of pride in the appearance of a dressing after the operation and he would stop to instruct his assistants as to a method of applying a bandage which at once would support and protect the parts and at the same time show to any observer that the work has been done with special thought and care. The direction of the layers, the superposition of the spicas, the permanency of the layers and the stability of the whole dressing were factors that were considered seriatim and they had to be learned and well learned by an assistant who expected to get on with his chief. When all was finished, a single pin held the dressing in place and it really looked so well that the friendns were favorably impressed by it and were likely to confine their comments to its interesting formation and thus spare the patient from hearing neighborhood gossip and the way a whole lot of people had died from the identical trouble that affected him. It was a good thing. The muslin and linen and flannel bandages have been sorely missed.

The advent of gauze bandages (and what frightfully flimsy stuff some of the hospitals furnish in the way of "gauze") made it possible for a man to lay a covering to a wound in almost any old way while the invention of a good adhesive plaster gave him something to make it stick even if the hairs of the skin subsequently rebelled when it was rapidly removed. It is true that this same gauze-plaster scheme enables one to put on lighter dressing very often and so it is a good thing, but there is still a field for the old methods and even in the application of gauze, there is a right way and ever so many wrong ones.

The book we have before us tells how to apply bandages. It tells it well and illustrates it by photographs that instruct at a glance. It goes thoroughly into the question and solves the problems. It will help many a very modern surgeon to make his dressings better not only for their classic character but for their comfort to his patients. The application of even flimsy gauze should not be hit or miss, but it should have evenness and supporting qualities that only the correct arrangement of the strips can give.

We commend the book to students and practitioners.

